# Supplementary material for: Existential aspects of fatherhood transition—A systematic qualitative review protocol using framework synthesis
Source: PLoS One. 2025 Dec 5;20(12):e0338056. doi: 10.1371/journal.pone.0338056 (PMC12680156; doi:10.1371/journal.pone.0338056)
Supplement: S1 File — (DOCX) [file pone.0338056.s001.docx]

## **S1 File. Example of Search string, EMBASE(OVID)**

| **#** | **Query** |
| --- | --- |
| 1 | father/ |
| 2 | father*.ti,ab,kf. |
| 3 | father child relation/ |
| 4 | father child relation.ti,ab,kf. |
| 5 | expectant father/ |
| 6 | mother/ |
| 7 | mother child relation/ |
| 8 | maternal.ti,ab,kf. |
| 9 | mother*.ti,ab,kf. |
| 10 | mother child relation*.ti,ab,kf. |
| 11 | expectant mother/ |
| 12 | exp parent/ |
| 13 | exp expectant parent/ |
| 14 | domestic partner/ |
| 15 | partner.ti,ab,kf. |
| 16 | spouse.ti,ab,kf. |
| 17 | parent*.ti,ab,kf. |
| 18 | paternal.ti,ab,kf. |
| 19 | couple*.ti,ab,kf. |
| 20 | 1 or 2 or 3 or 4 or 5 or 6 or 7 or 8 or 9 or 10 or 11 or 12 or 13 or 14 or 15 or 16 or 17 or 18 or 19 |
| 21 | exp childbirth/ |
| 22 | exp birth/ |
| 23 | exp live birth/ |
| 24 | exp life event/ |
| 25 | childbirth.ti,ab,kf. |
| 26 | transition.ti,ab,kf. |
| 27 | homebirth.ti,ab,kf. |
| 28 | birth.ti,ab,kf. |
| 29 | obstetric.ti,ab,kf. |
| 30 | parturition.ti,ab,kf. |
| 31 | labo?r.ti,ab,kf. |
| 32 | exp pregnancy/ |
| 33 | pregnan*.ti,ab,kf. |
| 34 | post?partum.ti,ab,kf. |
| 35 | postnatal.ti,ab,kf. |
| 36 | exp perinatal period/ |
| 37 | perinatal.ti,ab,kf. |
| 38 | prenatal.ti,ab,kf. |
| 39 | (becoming adj3 mother*).ti,ab,kf. |
| 40 | (becoming adj3 father*).ti,ab,kf. |
| 41 | (becoming adj3 parent*).ti,ab,kf. |
| 42 | life change.ti,ab,kf. |
| 43 | life event*.ti,ab,kf. |
| 44 | 21 or 22 or 23 or 24 or 25 or 26 or 27 or 28 or 29 or 30 or 31 or 32 or 33 or 34 or 35 or 36 or 37 or 38 or 39 or 40 or 41 or 42 or 43 |
| 45 | exp existentialism/ |
| 46 | exp meaning-making/ |
| 47 | exp spiritual care/ or exp spiritual well-being/ |
| 48 | exp religion/ |
| 49 | identity/ |
| 50 | existential*.ti,ab,kf. |
| 51 | life change.ti,ab,kf. |
| 52 | meaning mak*.ti,ab,kf. |
| 53 | spiritual*.ti,ab,kf. |
| 54 | human existence.ti,ab,kw. |
| 55 | perspective*.ti,ab,kf. |
| 56 | life world.ti,ab,kf. |
| 57 | psychological aspect.sh. |
| 58 | life event.ti,ab,kf. |
| 59 | identity.ti,ab,kf. |
| 60 | "internal conflict".ti,ab,kf. |
| 61 | reflect*.ti,ab,kf. |
| 62 | 45 or 46 or 47 or 48 or 50 or 51 or 52 or 53 or 54 or 55 or 56 or 57 or 58 or 59 or 60 or 61 |
| 63 | qualitative research/ |
| 64 | (("semi-structured" or semistructured or unstructured or informal or "in-depth" or indepth or "face-to-face" or structured or guide) adj3 (interview* or discussion* or questionnaire*)).ti,ab. |
| 65 | (focus group* or qualitative or ethnograph* or fieldwork or "field work" or "key informant").tw,kw. |
| 66 | experience*.ti,ab,kf. |
| 67 | 63 or 64 or 65 or 66 |
| 68 | 20 and 44 and 62 and 67 |
| 69 | limit 68 to (books or chapter or conference abstract or conference paper or "conference review" or data paper or editorial or erratum or letter or note or "preprint (unpublished, non-peer reviewed)" or "review" or short survey or tombstone) |
| 70 | 68 not 69 |
