# Supplementary material for: Existential aspects of fatherhood transition—A systematic qualitative review protocol using framework synthesis
Source: PLoS One. 2025 Dec 5;20(12):e0338056. doi: 10.1371/journal.pone.0338056 (PMC12680156; doi:10.1371/journal.pone.0338056)
Supplement: Attachment — Submitted filename: Response to Reviewers.docx [file pone.0338056.s004.docx]

**Dear editor and reviewers**

We address the comments from the editor and reviewers below one by one and in red colour, for each comment. We have also made the appropriate changes to the manuscript, and uploaded a revised edition, with all track changes highlighted in red.

We believe that the changes have improved our manuscript and hope you find it suitable for publication in PLOS ONE.

Best regards
on behalf of the authors

Marie Sejrsgaard

**Journal requirements:**

1. Please ensure that your manuscript meets PLOS ONE's style requirements, including those for file naming.

The required changes have been made to the formatting of the manuscript and the file naming.

2. Your ethics statement should only appear in the Methods section of your manuscript. If your ethics statement is written in any section besides the Methods, please move it to the Methods section and delete it from any other section. Please ensure that your ethics statement is included in your manuscript, as the ethics statement entered into the online submission form will not be published alongside your manuscript.

The ethics statement has been removed from the abstract and is now only to be found as a Subheading under the Method section (see line 144-147).

3. If the reviewer comments include a recommendation to cite specific previously published works, please review and evaluate these publications to determine whether they are relevant and should be cited. There is no requirement to cite these works unless the editor has indicated otherwise.

There was no such recommendation.

4. Please review your reference list to ensure that it is complete and correct. If you have cited papers that have been retracted, please include the rationale for doing so in the manuscript text, or remove these references and replace them with relevant current references. Any changes to the reference list should be mentioned in the rebuttal letter that accompanies your revised manuscript. If you need to cite a retracted article, indicate the article’s retracted status in the References list and also include a citation and full reference for the retraction notice.

Thank you, the reference list has been reviewed, and no changes have been made apart from adding four new references corresponding to the added discussion section.

**Additional Editor Comments:**

This is a well-reported systematic review protocol on an interesting and well-justified topic. Please find my recommendations below for improving the manuscript:
Thank you for this comment.

- Please clarify whether the protocol has been registered.
The protocol is registered in PROSPERO (see line 21-22).

- Please provide a rationale for choosing the four selected databases. Given the multidisciplinary nature of the topic, it is surprising that broad, multidisciplinary databases such as Web of Science and Scopus will not be used.
Thank you for this valuable feedback. After discussion and on your recommendation, we decided to include Web of Science on the list of databases to be searched.

- Please provide more details on how the framework synthesis approach will be applied, including the steps to be implemented.
Thank you for this opportunity. Please see the addition in line 127-139 to address this comment.

- The discussion section is missing. A complete discussion section needs to be included.
Some protocol articles include a discussion or elaboration on the introduction in the discussion. As the review is not completed it is subject to debate whether a discussion section is of value. We have chosen to expand on some of our reflections on the relevance and implications on the topic of this review in the discussion section (see line 148-157).

- No limitations are mentioned in the Strengths and Limitations section. Please address this.
Thank you for drawing this omission to our attention. The Strengths and Limitations section has been rewritten for a more coherent style, and to include some of the challenges of a review of this nature (see line 158-173).

- An explanation of how the strength of the body of evidence will be assessed is absent and should be provided.
Thank you for this comment. As our study is qualitative rather than quantitative, the concept of “strength of evidence” is not directly applicable. Instead, we assess the trustworthiness of the evidence, which is evaluated based on methodological rigor rather than statistical strength [1-3]. To ensure transparency and consistency, we will assess selectiveness of reporting by using the established appraisal tool; Critical Appraisal Skills Programme (CASP) checklist (see line 115-118). Additionally, we have added a level of meta reflection to the analysis process to enhance trustworthiness of the results (see line 139-142).

- Several typos are present throughout the manuscript and should be corrected.
We have submitted the manuscript to a third round of proofreading by a native English speaker.

1.	Malterud, K., V.D. Siersma, and A.D. Guassora, *Sample size in qualitative interview studies: guided by information power.* Qualitative health research, 2016. **26**(13): p. 1753–1760.

2.	Williams, V., A.-M. Boylan, and D. Nunan, *Qualitative research as evidence: expanding the paradigm for evidence-based healthcare.* BMJ evidence-based medicine, 2019. **24**(5): p. 168–169.

3.	Williams, V., et al., *Appraising qualitative health research-towards a differentiated approach.* BMJ Evid Based Med, 2022. **27**(4): p. 212–214.
